# Semantic integration of diverse data in materials science: Assessing Orowan strengthening

**DOI:** 10.1038/s41597-024-03169-4

**Published:** 2024-04-30

**Authors:** Bernd Bayerlein, Markus Schilling, Philipp von Hartrott, Jörg Waitelonis

**Affiliations:** 1 Bundesanstalt für Materialforschung- und prüfung (BAM), Unter den Eichen 87, Berlin, 12205 Germany; 2 Fraunhofer Institute for Mechanics of Materials IWM, Wöhlerstrasse 11, Freiburg, 79108 Germany; 3 Leibniz Institute for Information Infrastructure (FIZ Karlsruhe), Hermann-von-Helmholtz-Platz 1, Eggenstein-Leopoldshafen, 76344 Germany

**Keywords:** Mechanical engineering, Characterization and analytical techniques, Computational methods, Design, synthesis and processing

## Abstract

This study applies Semantic Web technologies to advance Materials Science and Engineering (MSE) through the integration of diverse datasets. Focusing on a 2000 series age-hardenable aluminum alloy, we correlate mechanical and microstructural properties derived from tensile tests and dark-field transmission electron microscopy across varied aging times. An expandable knowledge graph, constructed using the Tensile Test and Precipitate Geometry Ontologies aligned with the PMD Core Ontology, facilitates this integration. This approach adheres to FAIR principles and enables sophisticated analysis via SPARQL queries, revealing correlations consistent with the Orowan mechanism. The study highlights the potential of semantic data integration in MSE, offering a new approach for data-centric research and enhanced analytical capabilities.

## Introduction

In Materials Science and Engineering (MSE), material and process data are generated using a variety of different techniques. For instance, assessing a component’s degradation behavior at elevated temperatures necessitates data from both microstructural and mechanical analyses. The datasets resulting from these varied investigative techniques significantly differ in structure and format, posing a challenge for coherent integration and analysis^1,2^. Despite the increasing reliance on utilizing enhanced material and process data for progress^3^, much of this data remains in heterogeneous and unstructured formats^4^, leading to a fragmented and underutilized knowledge base, which is considered a loss of valuable resources^5,6^.

A central goal of digitalization in MSE is thus to achieve interoperability of material and process data from diverse sources, aligning with the FAIR principles^7^. Systematic data integration is expected to unlock valuable insights due to the intrinsic information and knowledge embedded within these data. Semantic Web technologies, particularly the development and application of ontologies, offer an effective approach to meet this challenge^8–11^. As defined by Gruber, an ontology is a “formal and explicit specification of a shared conceptualization”^12^. Ontologies facilitate context establishment, clear definition of meanings, formulation of relationships and rules, and linkages between data entities^13,14^. They are ideal for the semantic integration of heterogeneous data sources, enhancing their usability and accessibility for both human and machine processing^13,15,16^.

Ontologies are categorized into abstract, high-level forms such as top-level ontologies and mid-level ontologies, and specialized application ontologies. Application ontologies extend the concepts of mid-level ontologies and top-level ontologies to suit specific applications, inheriting their structural systematics crucial for interoperability^17,18^. Such an ontological construct enables the integration of data into a structured, semantic exchange format – the Resource Description Framework (RDF)^19–21^. The more data integrated into this semantic network or knowledge graph, the greater the accumulation of knowledge, enhancing the potential for artificial intelligence applications. For instance, improving natural language processing interpretability through ontological representations^22^, and increasing large language models accuracy^23^. Knowledge representation also provides a descriptive basis for pattern recognition^24^ and valuable information for decision making in areas such as machine learning and robotics^15,25,26^.

Despite the promising emerging applications and documented benefits in the literature^27^, MSE lacks practical, comprehensible examples demonstrating the steps of semantic data integration towards machine-processable knowledge representation^9,15^. Our work addresses this gap by developing a “good practice” Jupyter Notebook demonstrator for the MSE community. We employ the Orowan mechanism, a fundamental MSE theory that describes material strengthening through dislocations and precipitates^28^, as our use case. This demonstrator serves as a practical example of applying Semantic Web technologies in MSE, showing the use of ontologies and illustrating how valuable insights can be gained from heterogeneous data sources.

Our demonstrator addresses the following focal points:

- Methodical aggregation and structuring of two distinct, publicly available datasets from mechanical (tensile testing) and microstructural (dark-field transmission electron microscopy (DF-TEM)) characterizations of radial compressor wheels aged over several intervals.
- Development of an ontological framework using the PMD Core Ontology (PMDco) as a unifying mid-level ontology^29^.
- Seamless semantic integration of both datasets using specific application ontologies for tensile testing and DF-TEM image analysis data.
- Creation of a queryable knowledge graph as a proof-of-concept for semantic interoperability.
- Conducting targeted information queries to generate new insights via digital workflows, followed by enriching the knowledge graph.
- Demonstrating the process of indirect knowledge generation by uncovering hidden patterns in datasets through the querying of correlatable value pairs leading to the derived Orowan’s law.

## Results

In this section, we present the results of our research, which were obtained through information retrieval and analysis using the components of our Jupyter Notebook demonstrator, as referenced in^30^. The methods employed for ontological data representation and semantic data integration, within an ontological framework focused on the PMDco, are presented in detail in Section Ontological framework and Section Implementation of RDF graphs and ABox data instantiation.

By leveraging Semantic Web technologies, we establish meaningful links between two distinct datasets: the mechanical properties derived from tensile testing^31^ and the microstructural characteristics obtained from DF-TEM image analysis^32^ (see Section Tensile testing and DF-TEM imaging, respectively). This integration is achieved by embedding these datasets into a unified knowledge graph, where mid-level concepts serve as bridges, enhancing both data interoperability and comparability. The incorporation of these datasets into a knowledge graph is essential for developing a robust framework, which leverages SPARQL Protocol and RDF Query Language (SPARQL) queries for information retrieval and knowledge discovery^33^. Our approach enables exploration of the relationships between the mechanical and the microstructural properties of radial compressor wheels, offering deeper insights into their interdependence and aging behavior.

### Data retrieval, processing, and knowledge graph integration

This section describes our approach for managing and analyzing the data instantiated in Section Implementation of RDF graphs and ABox data instantiation, as well as for extending the knowledge graph with the newly derived results. The process involves three key steps: selective data retrieval using SPARQL queries (i), data processing through script-based workflows (ii), and integrating data outcomes back into the existing knowledge graph (iii), thereby enhancing it.

**Fig. 1:**
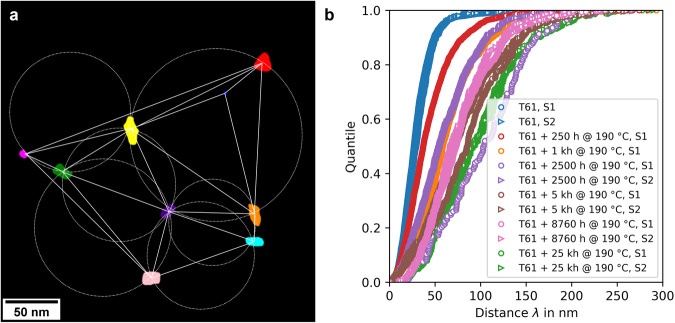
Analysis of precipitate distances using Delaunay triangulation. (**a**) Application of Delaunay triangulation to a DF-TEM image from the T61 + 2,500 h @ 190 °C, S1 dataset, illustrating the measured distances between segmented precipitates. (**b**) Displayed as a cumulative distribution function plot, this illustrates the range of precipitate distances within different material states, with labels S1 and S2 denoting the specific samples examined (refer to Table 1).
(iii) The calculated average precipitate distances, obtained by determining the mean values, are integrated into the existing knowledge graph. This involves creating a new class within the Precipitate Geometry Ontology (PGO), named pgo:AveragePrecipitateDistance, as a subclass of pgo:PrecipitateDistance. The computed data for the material states are then instantiated as instances of this new class, thereby enriching and expanding the knowledge graph.

(i) Initially, we extract specific information from the RDF dataset using precisely formulated SPARQL query that addresses the local triple store. For instance, Box 1 illustrates a designed query for processing microstructural data, retrieving details such as specimen images, material states, X and Y coordinates, and radii of precipitates from the DF-TEM image-based analysis dataset. The dataset relies on a conventional image processing procedure for analyzing precipitates, which includes several steps such as initially applying edge-preserving median filtering to the DF-TEM raw images, followed by manual thresholding for precipitate segmentation, as elaborated in Section DF-TEM imaging and referenced in^34^. Once this information is retrieved, the data is structured and prepared for further analysis, exemplifying the use of our locally managed RDF environment in drawing relevant insights from the data.
(ii) Next, the retrieved data is processed using a script-based workflow. Our primary focus is on determining the mean distances between precipitates and understanding how these mean distances vary across different material states, especially due to aging. For this purpose, we employ the Delaunay triangulation method^35^ to calculate precipitate distances for each material state and corresponding sets of images (see Table 1 for details). Each image’s precipitates are plotted using their X and Y coordinates. We use Delaunay triangulation to form triangles with precipitates as vertices, calculating the Euclidean distances between these vertices (Figure 1(a)). The resulting precipitate distances are then depicted in a cumulative distribution function plot (Figure 1(b)).

**Table 1:** Summary of DF-TEM image analysis dataset employed in this study.

Material State	Aging Temperature	Aging Time	Specimens	Images
T61	—	—	2	12
190 °C_250h	190 °C	250 h	1	11
190 °C_1000h	190 °C	1,000 h	1	12
190 °C_2500h	190 °C	2,500 h	2	17
190 °C_5000h	190 °C	5,000 h	2	23
190 °C_8760h	190 °C	8,760 h	2	19
190 °C_25000h	190 °C	25,000 h	2	21

Box 1 SPARQL query to retrieve, e.g., the X and Y coordinates of precipitates.

### Exploring Orowan strengthening through semantic data analysis

Building upon the semantic integration of the two distinct datasets outlined in Section Implementation of RDF graphs and ABox data instantiation and the newly obtained results from the Delaunay triangulation, we now apply these to explore the Orowan mechanism. The yield strength (*σ*_*ys*_) of alloys can be modeled as the cumulative effect of various mechanisms:

1 $${\sigma }_{ys}={\sigma }_{i}+{\sigma }_{ss}+{\sigma }_{p}+{\sigma }_{gs}$$

Here, *σ*_*i*_, *σ*_*ss*_, *σ*_*p*_, and *σ*_*gs*_ represent the intrinsic crystal, solid solution, precipitate strengthening, and grain size contributions, respectively. The precipitate strengthening component *σ*_*p*_ is typically expressed as the harmonic mean of contributions from dislocations shearing through shearable precipitates (particles) *σ*_*Friedel*_ and bowing between non-shearable precipitates *σ*_*Orowan*_. The contribution from dislocation bowing can in the idealized case be described as:

2 $${\sigma }_{Orowan}\propto \frac{G\cdot b}{\lambda }$$

In this equation, *G*, *b*, and *λ* are the shear modulus, the Burgers vector, and the edge-to-edge precipitate distance, respectively.

For our analysis, the material states exhibit significant variations primarily in the precipitate strengthening contribution *σ*_*p*_, with other factors in Equation (1) remaining constant. By correlating DF-TEM observed precipitate distributions, characterized by *λ*_*mean*_ (represented by pgo:AveragePrecipitateDistance in the ontology), with tensile test-derived *σ*_*ys*_ values, we aim to illustrate the contribution of *σ*_*Orowan*_ to the overall yield strength. It should be noted, that the Orowan stress *σ*_*Orowan*_ is defined for a single dislocation while the yield stress determined on a tensile test piece *R*_*p*02_ (represented by tto:ProofStrengthPlasticExtension) is the result of countless dislocation and an evaluation procedure prescribed by a standard. But it is common practice in materials science to correlate both.

**Fig. 2:**
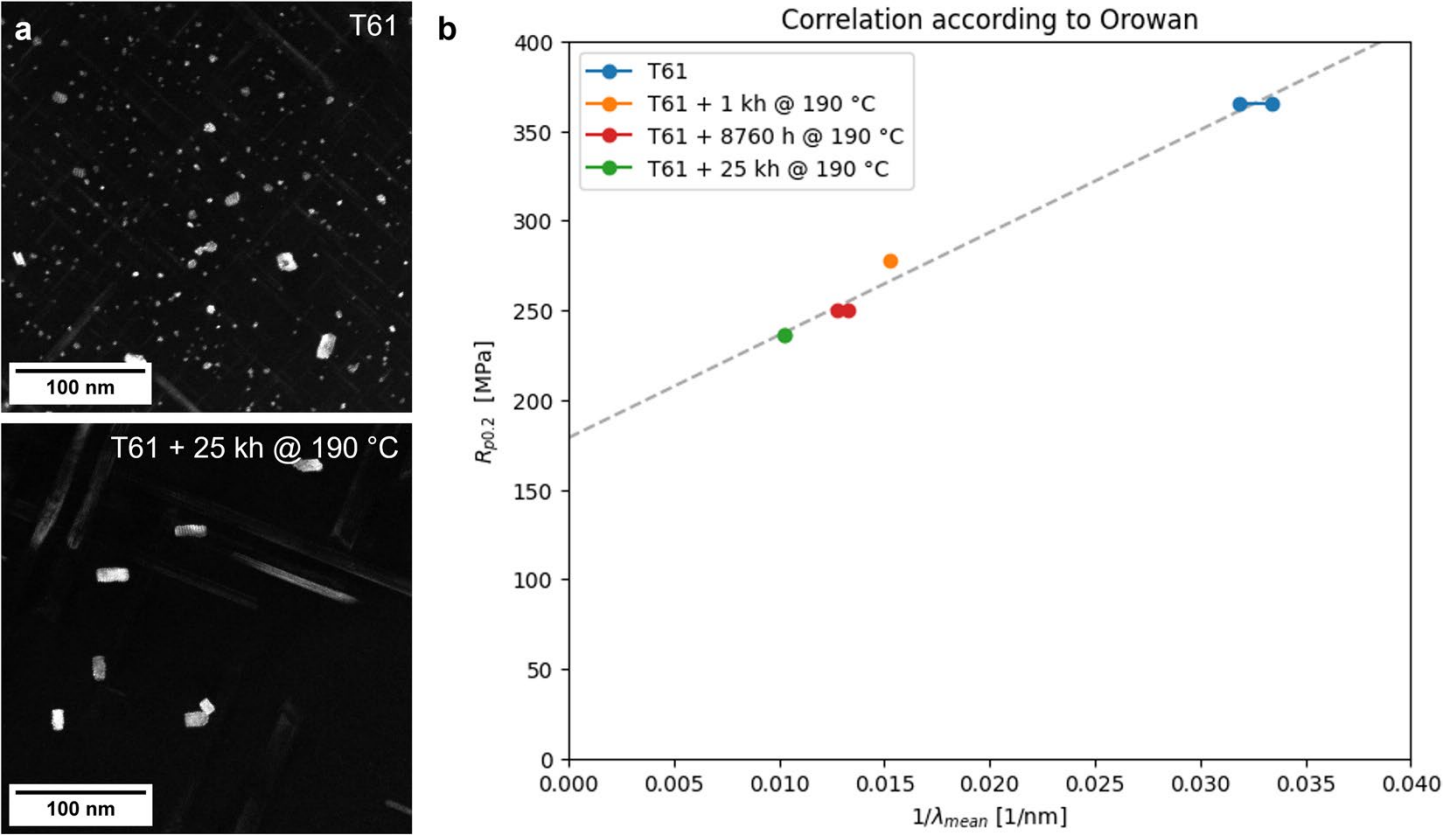
Impact of aging on material properties. (**a**) Displays examples of DF-TEM images for different material states: the upper image shows the T61 initial state, and the lower image depicts the state after aging at 190 ^°^C for 25,000 h. Notably, aging leads to coarsening, with precipitates becoming fewer and larger, thereby increasing the average precipitate distance. (**b**) Presents a plot derived from SPARQL query results, illustrating the dataset’s alignment with the expected trend of *σ*_*ys*_ ∝ 1/*λ*, as per Equation (2). It is important to note that tensile test data were available for only four material states, and the SPARQL query was employed to filter and identify six data point pairs for correlation across both mechanical and microstructural datasets.

Adhering to PMDco’s fundamental concepts enables the formulation of a SPARQL query that aggregate properties like materials yield strength *σ*_*ys*_ and average precipitate distance *λ* into a unified dataset (see Box 2). This unified dataset can be represented in a tabular format or further processed for visualization, as shown in Figure 2.

Box 2 SPARQL query for retrieving the correlatable proof strength plastic extension and their mean precipitate distance values.

## Discussion

### Establishing semantic interoperability

**Fig. 3:**
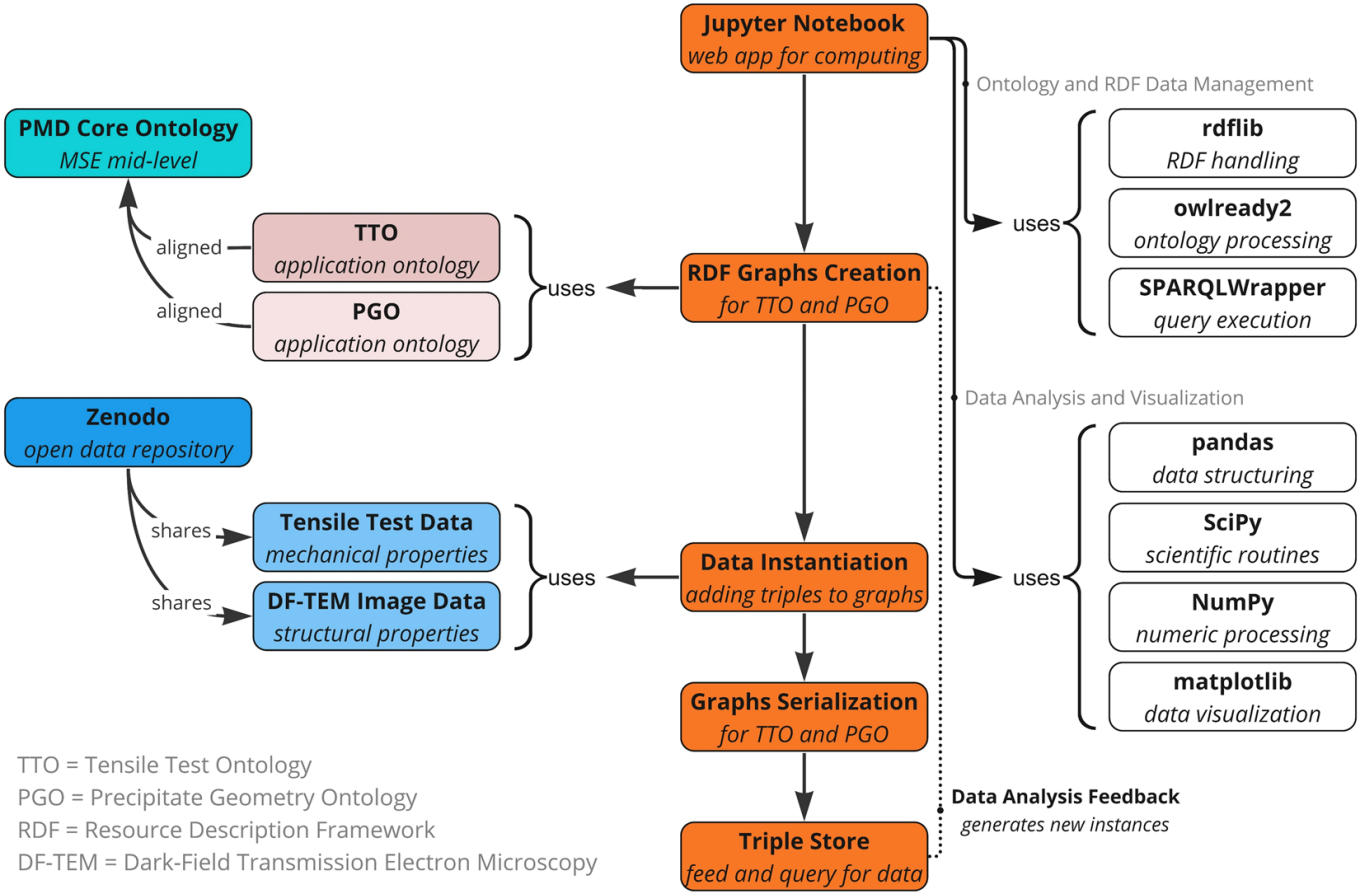
Key components of the demonstrator. This diagram outlines the key components and workflow of an ontology-centric data analysis framework within MSE. It encompasses the creation of RDF graphs, ontology alignment, data serialization, interactions with the local triple store, and the utilization of various libraries within the Jupyter Notebook environment. Our framework also involves analytical and visualization libraries for data processing, with a feedback loop for continuous development, and data sharing through Zenodo.

In this research, we have developed a demonstrator and described its functionality (Section Results and Section Implementation of RDF graphs and ABox data instantiation). The key components of this demonstrator are depicted in Figure 3. We employed an ontological framework that integrates the PMD Core Ontology (PMDco) with the Tensile Test Ontology (TTO) and the Precipitate Geometry Ontology (PGO) (Section Ontological framework). This setup enabled us to instantiate publicly available datasets from two different material characterization techniques sourced from Zenodo (see Data Availability statement).

The data from tensile testing and DF-TEM image analysis, while inherently different, have been transformed into interoperable RDF triples through the application of the PMDco, TTO and PGO. This transformation is facilitated by the interconnected concepts within the PMDco, which serve as bridges between the TTO and PGO entities. Consequently, a knowledge graph is established, allowing for SPARQL queries to retrieve instances across both TTO and PGO, filtered by material state (see Box 2). This demonstrates the principle of semantic interoperability and highlights the effectiveness of query filters in correlating diverse data sources.

Expanding upon this foundation, the creation of new classes and instances via SPARQL-driven data analysis systematically enriches our knowledge graph. This illustrates the scalable nature of our approach. However, in ontology-centric data management, any changes to the ontology structure must be managed carefully to avoid impacting its functionality. Developing a consistent mid-level ontology, aligned with a standard top-level ontology like the BFO^36^, can significantly enhance the interoperability of semantic data, extending well beyond the domain of MSE.

### Future perspectives in MSE knowledge representation

As already described in more detail in Section Introduction, the potential for ontology-based knowledge representation in MSE is substantial. Our research has established a sound basis for creating and expanding explicit knowledge representations in this field.

**Fig. 4:**
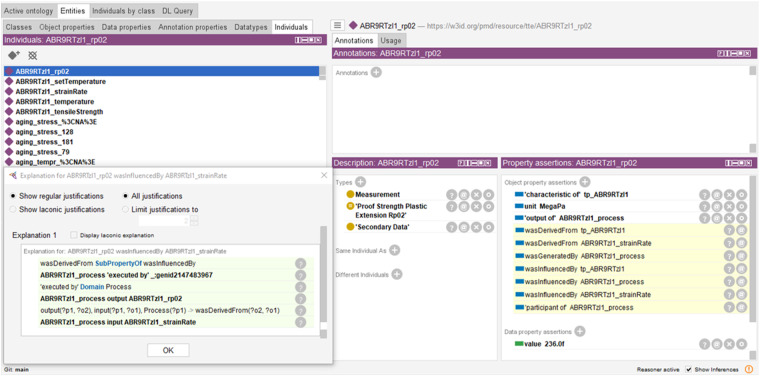
Exploring implicit MSE knowledge through reasoning. This screenshot demonstrates the implicit knowledge discovered by reasoning with Pellet in Protégé. It illustrates, for example, the inferred semantic connection between yield stress (the output of the process) and strain rate (a characteristic of the process) as applied in the experimental setup.

Looking ahead, the scope of MSE knowledge representation is expected to evolve beyond the current explicit modeling techniques. It will increasingly incorporate logical and computational methods to derive new insights and refine data curation practices. Central to this evolution is the role of reasoning. Our work has already provided a glimpse into the potential of reasoning (see Figure 4). In this context, for instance, the object property wasInfluencedBy denotes the inclusion of a specific and well-known strain rate in the dataset. As every process output is semantically connected to its inputs and characteristics, the ontology-based logic exposes the connection between yield stress (output of the process) and strain rate (characteristic of the process). This relationship is inherently semantic, which still has to be interpreted from a materials science perspective. As a result of our linked data processing in Protégé, the reasoning procedure successfully generated about 58,000 new triples. In general, reasoning, through the application of logical rules of ontologies, opens up new avenues for insight. Particularly intriguing is the automatic identification of inconsistencies, ensuring the relevance and accuracy of instantiated data. By integrating reasoning as a core method, we can develop more comprehensive and adaptable knowledge graphs, enhancing the MSE field’s capacity to manage and interpret complex data relationships.

Additionally, the use of languages and tools such as the Semantic Web Rule Language (SWRL)^37^ and Drools^38^ will provide further possibilities for optimizing knowledge representations. SWRL, which integrates the Web Ontology Language (OWL) with the Rule Interchange Format (RIF), lays a solid basis for complex inference and deduction processes crucial for understanding MSE data in depth^39^. This rule-based inference can significantly enhance the semantic richness of our approach. Furthermore, the integration of Drools as a rule engine marks a step forward in decision support and process automation, aligning with the specific data and contextual requirements of MSE.

### Limitations and outlook

In ontology-based data management, the reliance on SPARQL for query activities is a notable challenge. SPARQL’s complexity necessitates specific technical skills, posing a barrier to users unfamiliar with its syntax and the structure of RDF data^40,41^. To enhance user accessibility, future tools should focus on natural language processing capabilities, fostering an environment where queries are based more on intuitive human communication.

Interoperability of workflows and the adaptability of ontologies to diverse datasets also present critical areas for development in MSE. Current approaches are often tailored to specific datasets and limit broader application. Future efforts should aim at developing ontologies that are both robust and flexible, capable of handling varied data types and evolving information. Enhancing workflow interoperability is essential for managing the increasing volumes of data efficiently, thereby unlocking the full potential of semantic technologies in MSE for multidisciplinary research and insights.

### Summary

As a key outcome of our work, a demonstrator is provided that facilitates the interoperable linking of two publicly available datasets. These datasets, derived from distinct mechanical and structural characterization techniques of an aluminum alloy, are cohesively integrated using ontologies. The utilization of the PMDco enhances data usability and exemplifies practical effectiveness of semantic interoperability.

We have demonstrated the ability to query the constructed knowledge graph for specific information using SPARQL queries. This capability of generating new data from these queries, which then enriches the knowledge graph, underscores the dynamic and evolving nature of knowledge graphs. The continuous enrichment and expansion of the graph are key features of our approach.

Our work serves as a systematic and reproducible example of how data management and analysis in MSE can be aligned with FAIR principles, paving the way for future advancements. We also highlight key building blocks for semantic interoperability and create a basis which can be adapted and extended for different MSE applications.

In addition, our approach has shown the potential of Semantic Web technologies in uncovering hidden patterns across distinct datasets, making them potentially accessible and interpretable by machines. This opens new avenues for in-depth data analysis and insight generation, with machines playing a pivotal role in identifying trends and relationships that may not be immediately apparent to human researchers.

In summary, our study makes an important contribution to the field of MSE by providing tools and methods for semantic data integration, management, and analysis. These advancements enhance the field’s capacity to leverage data for advanced research and applications, marking a notable step forward in data-driven materials science exploration.

## Methods

### Sample material and states

Our study builds upon the prior research by Rockenhäuser *et al*.^34,42^, which focused on the coarsening processes of the S-phase in the aluminum alloy EN AW-2618A under elevated temperature conditions. In their experiments, the specimens underwent aging at 190 °C, extending up to 25,000 h. This process was followed by a detailed characterization of the evolved microstructure. The specimens were initially prepared in the T61 condition, a procedure that included solution annealing at 530 °C for 8 h, followed by rapid quenching in boiling water. Subsequent aging was conducted at 195 °C for a period of 28 h.

### Tensile testing

The tensile test dataset, accessible on Zenodo^31^, was compiled from tensile tests performed in accordance with the ISO 6892-1 standard^43^. These tests were conducted at a constant strain rate of 10^−4^ 1/s, utilizing B6 x 30 tensile test specimens, as specified by DIN 50125^44^.

### DF-TEM imaging

In our work, we utilized an image analysis dataset (see Table 1) from the aforementioned previous investigation by Rockenhäuser *et al*., accessible on Zenodo^32^. The dataset includes analysis data of dark-field transmission electron microscopy (DF-TEM) images focusing on S-phase precipitates within the aluminum matrix. The original DF-TEM images were captured with a JEM-2200FS TEM, operating at 200 kV, with specimens aligned along the [001] crystallographic direction. The DF-TEM images were provided in the dm3 format, a 16-bit raster format specifically designed for electron microscopy. This format includes vital metadata about the TEM procedure, such as details on the CCD camera, exposure time, and more.

To facilitate analysis, primarily the rod axis of the rod-shaped S-phase precipitates perpendicular to the image plane was imaged (The S-phase precipitates appear bright against a dark background). In addition, the precipitates were assumed to be cylindrical. The image-based evaluation was conducted on processed DF-TEM images using ImageJ software^45^. This process began with edge-preserving median filtering of the raw images, followed by manual thresholding for binarization. Such procedures enabled the exclusion of microstructural artifacts and horizontal rod-shaped precipitates from the analysis. The binarized image data facilitated the differentiation of the precipitates from the background. The dataset covers extensive evaluations of these images, with at least 300 precipitates analyzed for each material state. For an in-depth understanding of the materials, methodologies, and the software-based image analysis that yielded critical precipitate parameters like count, coordinates, area, and radius, readers are referred to the publications by Rockenhäuser *et al*.^34,42^.

### Software tools and libraries

The scripts for this work were developed within a Jupyter Notebook environment^46^. We utilized various Python^47^ -based libraries, including:

- The rdflib^48^ and Owlready2^49^ packages, crucial for semantic data processing and graph-based representations, facilitating semantic data integration.
- SPARQLWrapper for simplifying remote SPARQL query execution and results conversion^50^.
- NumPy^51^ and pandas^52^ for numerical operations.
- matplotlib for data visualizations^53^.
- SciPy for advanced data manipulation and visualization tasks, including Delaunay triangulation^54^.
- The Protégé ontology editor^55^, supporting OWL 2 Web Ontology Language^56^, for ontology design and Pellet for reasoning^57^.

### Ontologies and GitHub integration

The PMD Core Ontology (PMDco), Version 2.0.7, serves as the upper-level ontology, providing bridging mid-level concepts crucial for achieving semantic interoperability^29,58^.

For the representation of tensile tests of metals at room temperature, aligned with ISO 6892-1:2019-11^43^, we employed the Tensile Test Ontology (TTO)^59,60^.

The Precipitate Geometry Ontology (PGO)^61^ is used for representing microstructural data derived from DF-TEM image analysis.

QUDT entities were incorporated for units of measure^62^.

GitHub is used for publishing, maintaining, and developing these ontologies along the associated scripts^63^.

### Ontological framework

This work utilizes an ontological framework structured around PMDco, a mid-level ontology specifically developed for MSE. PMDco is a foundational reference for semantically bridging more specialized MSE application ontologies, namely in our work, the TTO and the PGO. The TTO facilitates the systematic representation of tensile test data, while the PGO enables representation of microstructural data. Together these interconnected ontologies establish a semantic network that standardizes the representation, querying, and analysis of distinct datasets.

#### Achieving semantic interoperability with the PMDco

The PMDco plays a pivotal role in semantically interlinking the TTO and the PGO. While each application ontology addresses specific domain-related classes, the PMDco offers broader MSE concepts, ensuring the application ontologies are embedded in a structured, extendable system. This framework allows for the addition of application-specific classes, crucial for representing diverse data sources coherently, thereby enhancing semantic interoperability.

#### Standardizing tensile test data with the TTO

Based on the PMDco, the Tensile Test Ontology (TTO) was designed to provide a structured vocabulary for tensile test data. Thereby, the test standard-compliant ontological representation ensures this data to be interoperable, transparent, reliable, and reproducible. Hence, the TTO is crucial in the semantic integration of tensile test data, particularly in this work concerning radial compressor wheel samples. Aligned with ISO 6892-1:2019-1 standard for tensile testing of metals at room temperature (see Section Ontologies and GitHub integration), the terminological box (TBox) of the TTO ensures a standardized approach for data integration. Therein, a comprehensive number of classes is included to specifically annotate data on characteristic values obtained from a tensile test, such as, e.g., yield strength (*R*_*m*_), proof strength, plastic extension (*R*_*p*_), and strain rate ${\dot{e}}_{{L}_{e}}$. Furthermore, semantic relationships are also implemented that enable logical reasoning and thus, lead to improved data interpretation capabilities (see Section Future perspectives in MSE knowledge representation). Overall, the TTO harmonizes tensile test data, which often varies in structure and format, into uniform RDF triples, thus improving data comparability and method reproducibility by incorporating essential contextual information, such as metadata and provenance^59^.

#### Integrating microstructural data with the PGO

**Fig. 5:**
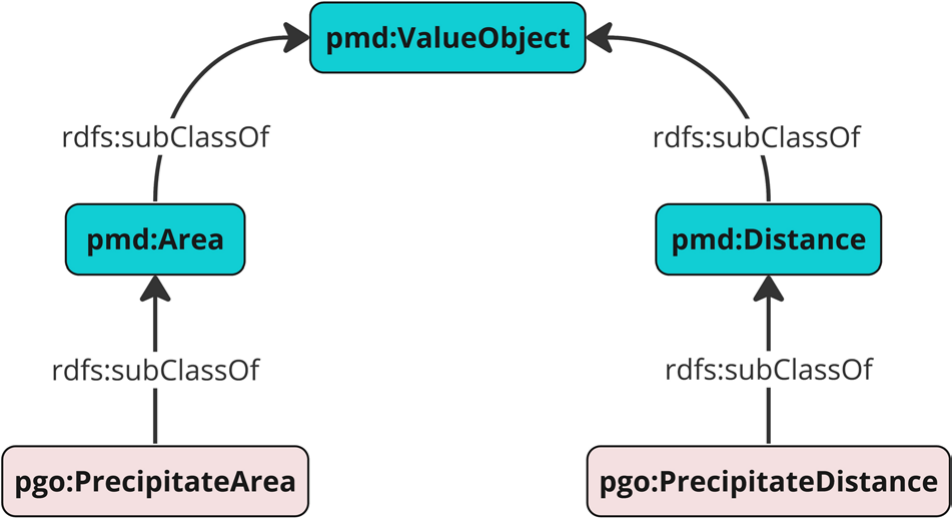
Class hierarchy in the Precipitate Geometry Ontology (PGO). This figure illustrates PGO’s application-specific subclasses, such as pgo:PrecipitateArea and pgo:PrecipitateDistance, extending from PMDco’s general pmdco:ValueObject class via pmdco:Area and pmdco:Distance, respectively.

Integration of microstructural data from DF-TEM image analysis is accomplished using the Precipitate Geometry Ontology (PGO). Designed to extend PMDco’s class structure, the PGO introduces specific subclasses for a more detailed representation of precipitate data. These subclasses, such as pgo:PrecipitateArea and pgo:PrecipitateDistance, enhance the granularity of the data representation, facilitating the construction of an informative KG. Figure 5 illustrates an exemplary subclass hierarchy of the PGO in relation to the broader PMDco structure.

### Implementation of RDF graphs and ABox data instantiation

In a Jupyter Notebook environment, we initiate the process by importing ontologies (i), constructing RDF graphs (ii), and instantiating ABox (Assertion Box) data (iii). These steps are supported by specific Python libraries, as detailed in Section Software tools and libraries.

**Fig. 6:**
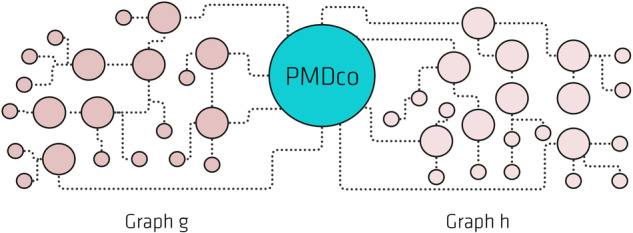
Facilitating semantic integration via the PMDco. This diagram schematically depicts the semantic connections between two application ontologies, graphs “g” and “h”. The PMDco is a mid-level ontology, providing general MSE concepts crucial for bridging these ontologies.

**Fig. 7:**
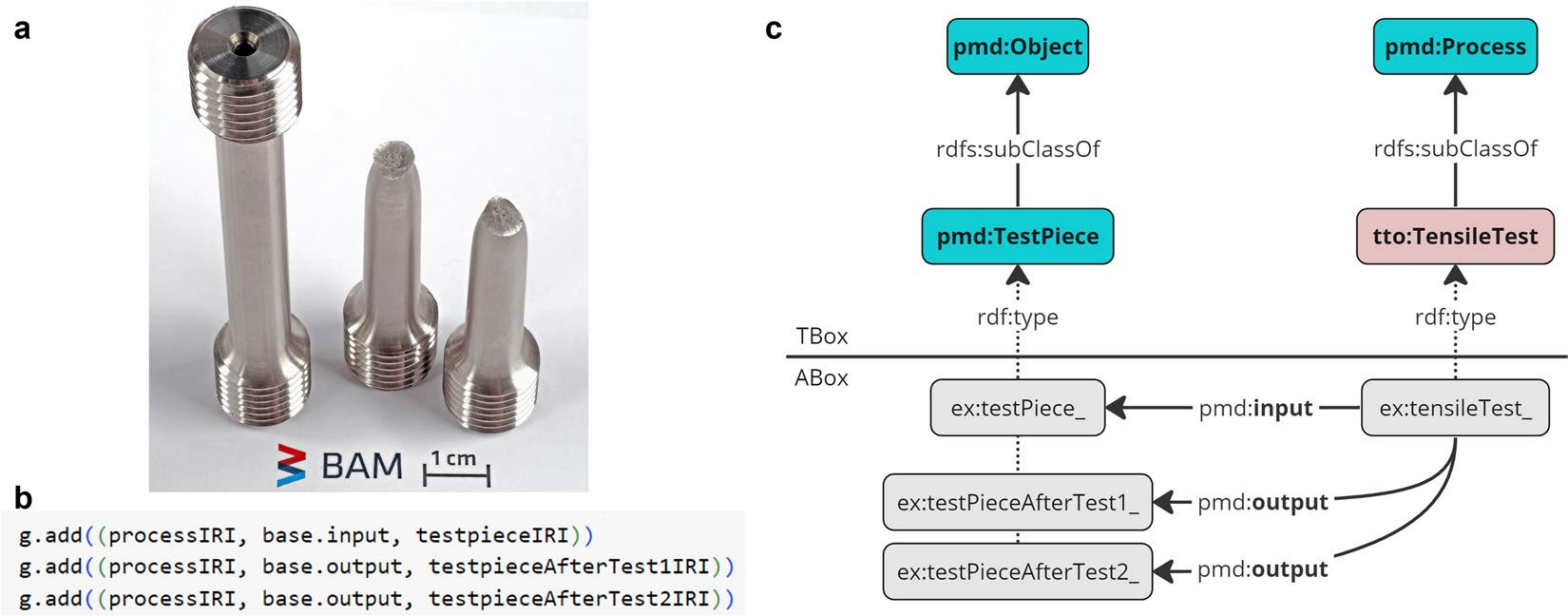
Tensile test pieces and RDF integration. (**a**) Tensile test pieces before and after testing, showing intact and fractured states. (**b**) RDF triple integration in tensile testing, illustrated by g.add((processIRI, base.input, testpieceIRI)). In this context, the tensile test piece (denoted by testpieceIRI) is the input (identified by base.input) and output (identified by base.output) for the tensile test process (represented by processIRI). (**c**) Schematic of PMDco’s role in connecting TTO entities in tensile testing. Instances such as ex:testPiece_, ex:testPieceAfterTest1_, and ex:testPieceAfterTest2_ are categorized under pmd:TestPiece class using the rdf:type property.
(iii) Subsequently, the tensile test and DF-TEM datasets are instantiated within these graphs. ABox instances are created based on the TBox classes and properties, utilizing RDF triples (subject, predicate, object) for data serialization. This process establishes the relationships between entities, with each assigned a unique Internationalized Resource Identifier (IRI) for identification (refer to Figure 7), thereby enabling advanced data querying and analysis^64^.

(i) Initially, the PMDco and the application ontologies, including the TTO and PGO TBoxes, in Turtle format (ttl), are imported into the Notebook environment.
(ii) We then proceed to construct distinct RDF graphs for integrating the datasets: “g” for tensile test data and “h” for DF-TEM image analysis data. Both graphs are aligned with the PMDco, providing an ontological framework for semantic interoperability (see Figure 6). Graph “g” encompasses tensile test entities such as measurement values, as well as the initial test pieces (input) and the resulting fractured halves (output) (see Figure 7(a)). It comprehensively details mechanical properties like yield and proof strength, environmental conditions during testing, and descriptions of the material states. Graph “h” focuses on microstructural data, representing precipitate characteristics such as the coordinates, areas, and distances. The RDF representations bridge mechanical and microstructural data, enabling flexible correlations within a unified semantic network.

## Data Availability

The tensile test dataset^31^ and the TEM microstructural analysis dataset^32^, both hosted on Zenodo, are integral to our study. The tensile test dataset delivers comprehensive results from tests conducted on EN AW-2618A aluminium alloy. The TEM dataset provides detailed quantitative analysis of the microstructure of the same alloy, with a particular emphasis on examining the S-phase *A**l*_2_*C**u**M**g* parameters. The RDF graph data, resulting from the semantic integration of these two datasets, is available on GitHub. Access to the Tensile test RDF graph data, the DF-TEM RDF graph data, the DF-TEM RDF graph data addition, and the demo-orowan RDF graph:• Tensile test RDF graph data• DF-TEM RDF graph data• DF-TEM RDF graph data addition• demo-orowan RDF graph data

## Code availability

The script and the ontologies utilized in this study are openly accessible and can be obtained from the following GitHub repositories:• demo-orowan• PMD Core Ontology (PMDco)• Tensile Test Ontology (TTO)• Precipitate Geometry Ontology (PGO)
